# Social Media Use among Orthopedic Trauma Fellowship-trained Surgeons

**DOI:** 10.1055/s-0044-1779314

**Published:** 2024-03-21

**Authors:** Haley E. Smith, Colin K. Cantrell, Cody J. Goedderz, Michelle L. Wiese, Ramiz Memon, Joel C. Williams

**Affiliations:** 1 Departamento de Cirurgia Ortopédica, Northwestern Feinberg School of Medicine, Chicago, IL, Estados Unidos; 2 Northwestern Feinberg School of Medicine, Chicago, IL, Estados Unidos; 3 William Beaumont School of Medicine, Oakland University, Rochester, MI, Estados Unidos; 4 Departmento de Cirurgia Ortopédica, Rush University Medical Center, Chicago, IL, Estados Unidos

**Keywords:** orthopedics, trauma, social media, fellowships and scholarships

## Abstract

**Objective:** To quantify the use of social media platforms by orthopedic traumatologists with an emphasis on demographic, practice-based, and regional differences.

**Materials and Methods:** Using the Orthopaedic Trauma Association (OTA) membership database, online searches were performed to identify professional profiles on numerous social media platforms. This presence was then quantified by a cumulative social media score which was correlated to the demographic information collected.

**Results:** In total, 1,262 active fellowship-trained orthopedic traumatologists were identified. Surgeons practicing in an academic setting were found to be more likely to use numerous social media platforms and to present an overall greater social media score than those in private practices. No significant differences in use were found based on practice region.

**Conclusion:** Social media platforms are currently underused by orthopedic traumatologists.

**Level of Evidence:** IV.

## Introduction

In the last decade, the rapid expansion of social media has made it a powerful and influential medium in personal and professional life. In 2021, the global population who is active on social media reached 4.2 billion people. 1 Inevitably, the popularity of social media has impacted its use in healthcare settings. Recent studies 2 have shown that more than 75% of patients are researching their physicians, hospitals, and conditions prior to selecting a provider. Through the use of social media, surgeons have the opportunity to educate, communicate, and market themselves to patients at a free or minimal cost. Additionally, the rapid dissemination of information via social media platforms provides a digital-user interactive space for collaboration, research, and education.

Numerous studies have examined the use of social media platforms by physicians. The rate of engagement varies extensively based on provider specialty, age, and practice setting. Surgical subspecialities, including urology and plastic surgery, have found that more than 50% of providers have at least 1 social media account. 3 4 The studies 3 5 examining the use of social media by orthopedic surgeons have demonstrated underutilization. To our knowledge, the present is the first study on social media use among orthopedic traumatologists. The purpose is to quantify and analyze the use of social media among fellowship-trained orthopedic trauma surgeons.

## Materials and Methods

### Identification of Surgeons and Demographic Data

The present study was exempt from institutional review board approval. The information was gleaned from publicly available data. The membership database of the Orthopaedic Trauma Association (OTA) was queried to identify active fellowship-trained orthopedic trauma surgeons. The senior author is an active member of the OTA and has access to this membership database. The categories of *active* , *candidate* , and *clinical* were queried for members. Once this list was compiled, a Google (Mountain View, CA, United States) search was performed using member name + “orthopedic trauma” or “orthopedic surgeon” to determine if the member was an actively practicing fellowship-trained orthopedic trauma surgeon. Only the first page of results for each search was viewed. Information stating that the surgeon was trauma fellowship-trained or listed fellowship location was used to determine inclusion. Members who did not meet these criteria or lacked sufficient information to determine this were excluded.

The demographic data collected for each member included name, practice type, and practice location. Practice locations were further grouped among five regions, four of which are in the United States: Northeast, Midwest, West, South, and Canada. A full listing of states within each region is located in Table 1 .

**Table 1:** Regions by US States

Regions			
West	Midwest	South	Northeast
WA	ND	TX	MD
OR	SD	MS	DC
CA	NE	AR	PA
ID	KS	LA	DE
NV	OK	KY	NJ
UT	MN	TN	NY
AZ	IA	MO	CT
MT	WI	AL	MA
WY	IL	FL	RI
CO	MI	GA	VT
NM	IN	SC	NH
AK	OH	NC	ME
HI	WV	VA	
		Puerto Rico	

### Social Media Analysis

A social media analysis was adopted from Narain et al. 3 and Lander et al. 5 Seven separate platforms were assessed for member presence: Facebook, X, Instagram, LinkedIn, ResearchGate, YouTube, and a professional website. This was performed following a standard method with the same search criteria as the previous Google search on each individual platform. Personal or private profiles which were not primarily related to the surgeon's professional work were excluded. Practice websites must have been created for that individual surgeon to be included, with websites for a department or for multiple providers of a group excluded.

Once the platform profiles were identified, active use was determined on Facebook, X, Instagram, and YouTube. This is defined as activity on the profile in the previous six months. The three other platforms (LinkedIn, ResearchGate, and the professional website) were not assessed for activity because they are not based on current postings or activity. An overall social media score was then calculated by tallying up the number of profiles each member had present on our search. For the platforms that were assessed for activity, only active profiles were calculated as present in the social media score.

### Statistical Analysis

Surgeon demographics were compared. Categorical variables are reported as frequency and percentages, and they are compared using Chi-squared test. Continuous variables are reported as mean with standard deviations (SD) for normally-distributed variables. These are compared with the Student *t* -test or analysis of variance (ANOVA) tests. The alpha level was set at 0.05. All data and statistical analyses were performed using the JMP Pro software, version 16.0 (SAS Institute Inc., Cary, NC, United States).

## Results

### Demographics

In total, 1,262 members were identified as active orthopedic trauma fellowship-trained surgeons: 90% (1130) of them members were male, and 567 (44.9%) members practice in an academic setting, while the other 695 (55.1%) were identified as practicing in a private setting. The southern region contained the most members: 400 (31.7%). Full demographic data are in Table 2 .

**Table 2:** Surgeon demographics

Variable	Surgeons ( *n* = 1,262)
**Sex**	
Female	132 (10.5%)
Male	1,130 (89.5%)
**Practice setting**	
Academic	567 (44.9%)
Private	695 (55.1%)
**Region**	
Canada	62 (4.9%)
Midwest	272 (21.5%)
Northeast	252 (20.0%)
South	400 (31.7%)
West	276 (21.9%)

### Social Media Platforms

Use varied among social media platforms. Instagram was the least prevalent, with only 34 (2.7%) members maintaining an account, and 23 (1.8%) active accounts. Regarding currently-active accounts, X was the most prevalent, with 91 (7.2%) accounts, 58 (4.6%) of which were deemed active. LinkedIn was the most used platform, with more than half (54%) of the members identified with accounts. The average social media score was of 1.24 ± 1.05 (range: 0 to 6). No member received the perfect social media score of 7. Full details regarding social media use are located in Table 3 .

**Table 3:** Social media accounts by region

Platform	All ( *n* = 1,262)	Canada ( *n* = 62)	Midwest ( *n* = 272)	Northeast ( *n* = 252)	South ( *n* = 400)	West ( *n* = 276)	*p* -value
Facebook	59 (4.7%)	0 (0%)	18 (6.6%)	8 (3.2%)	20 (5.0%)	13 (4.7%)	0.15
Active	30 (2.4%)	0 (0%)	8 (2.9%)	4 (1.6%)	12 (3.0%)	6 (2.2%)	0.52
X	91 (7.2%)	6 (9.7%)	21 (7.7%)	21 (8.3%)	27 (6.8%)	16 (5.8%)	0.73
Active	58 (4.6%)	4 (6.5%)	12 (4.4%)	17 (6.7%)	16 (4.0%)	9 (3.3%)	0.33
Instagram	34 (2.7%)	1 (1.6%)	8 (2.9%)	8 (3.2%)	11 (2.8%)	6 (2.2%)	0.93
Active	23 (1.8%)	1 (1.6%)	4 (1.5%)	5 (2.0%)	8 (2.0%)	5 (1.8%)	0.99
LinkedIn	683 (54.1%)	37 (59.7%)	145 (53.3%)	142 (56.3%)	218 (54.5%)	141 (51.1%)	0.65
ResearchGate	290 (23.0%)	20 (32.3%)	51 (18.8%)	71 (28.2%)	80 (20.0%)	68 (24.6%)	0.016
YouTube	433 (34.3%)	15 (24.2%)	96 (35.3%)	102 (40.5%)	137 (34.3%)	83 (30.1%)	0.056
Website	44 (3.5%)	1 (1.6%)	9 (3.3%)	6 (2.4%)	13 (3.3%)	15 (5.4%)	0.31
Social media score	1.24 ± 1.05	1.26 ± 0.85	1.19 ± 1.03	1.38 ± 1.11	1.21 ± 1.04	1.18 ± 1.05	0.21

### Regional Social Media Use

When broken down into the four regions of the United States plus Canada, only ResearchGate produced statistically significant differences in terms of use ( *p* = 0.016). Canada led all regions, with 32% of surgeons using this platform, with the northeastern United States following with 28%. Full details on social media platform use are in Table 3 .

### Practice Type and Social Media Use

Variations in social media use among surgeons in academic and private practices we observed in a few different platforms. Academic surgeons were more likely to use an active X account ( *p* = 0.0001), a LinkedIn page ( *p* = 0.0055), a ResearchGate page ( *p* < 0.0001), and YouTube ( *p* = 0.0002). Private surgeons were only more likely to use a professional website ( *p* = 0.0008). Academic surgeons presented a higher average social media score (1.43) when compared with private surgeons (1.08) ( *p* < 0.0001) ( Table 4 ).

**Table 4:** Social media accounts by practice type

Platform	All ( *n* = 1,262)	Academic ( *n* = 567)	Private ( *n* = 695)	RR (95%CI)	*p* -value
Facebook	59 (4.7%)	20 (3.5%)	39 (5.6%)	0.627 (0.37–1.06)	0.084
Active	30 (2.4%)	9 (1.6%)	21 (3.0%)	0.52 (0.24–1.14)	0.1
X	91 (7.2%)	55 (9.7%)	36 (5.2%)	1.87 (1.25–2.8)	0.0025
Active	58 (4.6%)	42 (7.4%)	16 (2.3%)	3.21 (1.83–5.65)	0.0001
Instagram	34 (2.7%)	17 (3.0%)	17 (2.4%)	1.01 (0.99–1.02)	0.55
Active	23 (1.8%)	13 (2.3%)	10 (1.4%)	1.59 (0.7–3.6)	0.27
LinkedIn	683 (54.1%)	331 (58.4%)	352 (50.6%)	1.15 (1.04–1.28)	0.0055
ResearchGate	290 (23.0%)	184 (32.5%)	106 (15.3%)	2.12 (1.72–2.63)	< 0.0001
YouTube	433 (34.3%)	225 (39.7%)	208 (29.9%)	1.33 (1.14–1.55)	0.0002
Website	44 (3.5%)	8 (1.4%)	36 (5.2%)	0.27 (0.13–0.58)	0.0008
Social media score	1.24 ± 1.05	1.43 ± 1.10	1.08 ± 0.97		< 0.0001*

Abbreviations: 95%CI, 95% confidence interval; RR, relative risk.

Note: * *t* -test.

## Discussion

The present is the first study to examine the use of social media among orthopedic traumatologists. We found that orthopedic traumatologists underuse social media, and use did not significantly vary among practice location. There was a variation in use among academic and private-practice surgeons, with academic-practice surgeons more likely to use numerous platforms and have higher overall social media scores.

Historically, patients relied on other physicians' recommendations and word of mouth for specialty and surgical referrals. In the last decade, there has been a 10-fold increase in the use of social media, with ∼ 80% of users reporting searching the internet for physicians, medical conditions, or treatment options. 2 As the population increasingly relies on social media and patient satisfaction scores to make their decisions on healthcare providers, marketing and patient engagement strategies will need to adapt to involve these new platforms.

A review of social media use in plastic surgery found that a single post can generate 10 to 12 reposts, resulting in a broader marketing reach. 6 7 Research within other orthopedic subspecialties has demonstrated that a more robust online presence was associated with higher patient satisfaction scores. 8 9 In the present study, we found that the average social media score among traumatologists was of 1.24 ± 1.05 (range: 0 to 6). Donnally et al. 8 reported that hand surgeons with a social media score lower than 3 received lower Healthgrades scores when compared with those that had a social media index higher than 3. This highlights the potential for orthopedic traumatologists to improve their patient-satisfaction and engagement through social media. Further research is needed to understand the impact of social media on orthopedic practice growth and patient satisfaction within orthopedic traumatology.

Further emphasizing the increasing relevance of social media to the orthopedic practice, the American Academy of Orthopaedic Surgeons (AAOS) as well as the *Journal of Orthopaedic Trauma* have published guidelines and recommendations to build social media presence. 2 9 10 These resources encourage physicians to engage in social media to maximize opportunities or personal and professional development. The guidelines explain usage basics for social media novices and provide recommendations on maintaining the professional standard in the digital space.

In the present study, the use of the most popular social medial platforms by the orthopedic surgeons was low: 34.3% of them had an active YouTube presence, 2.4% had an active Facebook page, and 1.8% had an active Instagram account. These figures represent a substantially lower rate of use compared with the US adult social media usage averages of 73% for YouTube, 69% for Facebook, and 37% for Instagram. 1 3 Underutilization of social media has also been reported in other fields of orthopedic surgery. Of 987 pediatric orthopedic surgeons, 33% had a YouTube presence and 14% had a professional Facebook Page. 5 In shoulder elbow surgery, 12.9% of surgeons surveyed had an active YouTube presence and 10.4% had a professional Facebook page. 3 The low social media use among orthopedic traumatologists represents a missed opportunity for practice and professional development.

Practice setting frequently impacts overall practice patterns, especially regarding marketing and patient referrals. While academic-practice orthopedic traumatologists are using social media more extensively than their peers in private practices in the present study, this contradicts many other studies on social media use in healthcare, which have found an increased use in private practice. 4 6 11 This may be due to a decreased need for a referral base and promotion in traumatology compared with other surgical practices. Within the field of orthopedic trauma, the increased use of social media in an academic setting may be related to its use primarily as an educational tool rather than a patient-recruitment medium. Studies have demonstrated that academic productivity has been linked to increased social media use. 12 13

Low social media engagement among orthopedic traumatologists may stem from a variety of reasons. Surgeons could view social media is inefficient, especially if they lack experience in the use the various platforms. 14 Additionally, there may be less motivation to utilize social media in trauma relative to other elective orthopedic subspecialties that rely more heavily on patient selection of a provider and a strong referral basis. 12 The use of social media in healthcare is not without controversy or concern. Surgeons may be concerned about the potential for violations of the Health Insurance Portability and Accountability Act (HIPAA) of 1996, professionalism, or ethical standards. Furthermore, there may also be worries regarding the ability to maintain boundaries in patient-physician contact. 2 There may also be low use due to concerns related to personal reputation and professionalism. More than 60% of recruiters check the social media accounts of potential employees. 10 Overall, social media may provide benefits to the practice of orthopedic trauma, including professional networking, education, and patient engagement. However, it is imperative that orthopedic traumatologists adhere to a strict standard of professionalism when integrating social media into their clinical practice.

There are several limitations to the present study. First, we only analyzed OTA members with publicly-available information pertaining to their fellowship training who maintain public-domain social media accounts. Therefore, our results may not be completely representative of social media use among traumatologists as a whole. Additionally, the present study was observational in nature and represents a single point in time. Social media use is dynamic, and the single point in time may under or overestimate utilization. Further research is needed to understand the type of content and materials that facilitate education, networking, and public engagement. Lastly, in the present study we were not able to comment on the barriers or concerns regarding social media use among orthopedic traumatologists.

## Conclusion

Social media platforms are currently underused by orthopedic traumatologists. Additional research is warranted to understand the barriers to social media use within the OTA community.
