# Supplementary material for: Social Media Use among Orthopedic Trauma Fellowship-trained Surgeons
Source: Rev Bras Ortop (Sao Paulo). 2024 Mar 21;59(1):e119–24. doi: 10.1055/s-0044-1779314 (PMC10957270; doi:10.1055/s-0044-1779314)
Supplement: Related texts

# Research article: Uso de redes sociais por cirurgiões com treinamento especializado em trauma ortopédico

**Keywords:** ortopedia, trauma, redes sociais, bolsas de estudo

## Abstract

**Objetivo:** Quantificar o uso de plataformas de rede social por traumato-ortopedistas, com ênfase nas diferenças demográficas, regionais e de tipo de prática clínica.

**Materiais e Métodos:** Utilizando o banco de dados de membros da Orthopaedic Trauma Association (OTA), foram realizadas pesquisas *on-line* para identificar perfis de profissionais em diversas plataformas de rede social. Esta presença foi quantificada por uma pontuação cumulativa de redes sociais, que foi correlacionada com as informações demográficas coletadas.

**Resultados:** Foram identificados 1.262 profissionais com treinamento especializado em trauma ortopédico. Observou-se que os cirurgiões que atuam em ambiente acadêmico têm maior probabilidade de usar diversas plataformas de rede social e apresentam pontuação geral maior em redes sociais do que aqueles que atuam em consultório particular. Não foram encontradas diferenças significativas quanto ao uso de redes sociais com base na região de atuação.

**Conclusão:** Atualmente, as plataformas de rede social são subutilizadas pelos traumato-ortopedistas.

**Nível de Evidência:** IV.

## Introdução

Na última década, a rápida expansão das redes sociais tornou-as um meio poderoso e influente na vida pessoal e profissional. Em 2021, a população global ativa nas redes sociais atingiu 4,2 bilhões de pessoas. 1 Inevitavelmente, a popularidade das redes sociais influenciou sua utilização em ambientes de saúde. Estudos recentes 2 mostraram que mais de 75% dos pacientes pesquisam seus médicos, hospitais e doenças antes de escolher um profissional ou instituição. Por meio das redes sociais, os cirurgiões têm a oportunidade de educar, comunicar-se e promover-se para os pacientes de forma gratuita ou com custo mínimo. Além disso, a rápida disseminação de informações pelas plataformas de rede social gera um espaço interativo de colaboração, pesquisa e educação para o usuário digital.

Diversos estudos analisaram o uso médico de plataformas de rede social. A taxa de envolvimento é bastante variável conforme a especialidade, a idade e o ambiente de atuação do profissional. Em subespecialidades cirúrgicas, como urologia e cirurgia plástica, mais de 50% dos profissionais têm pelo menos uma conta de rede social. 3 4 Os estudos acerca do uso das redes sociais por cirurgiões ortopédicos demonstraram subutilização. 3 5 Pelo que sabemos, este é o primeiro estudo a examinar o uso de redes sociais entre traumato-ortopedistas. O objetivo foi quantificar e analisar o uso das redes sociais entre cirurgiões com treinamento especializado em traumatologia ortopédica.

## Materiais e Métodos

## Identificação dos Cirurgiões e Dados Demográficos

Este estudo foi isento da aprovação do comitê de ética institucional. As informações foram coletadas a partir de dados públicos. O banco de dados de membros da Orthopaedic Trauma Association (OTA) foi consultado para identificar cirurgiões com treinamento especializado em traumatologia ortopédica. O autor sênior é membro ativo da OTA e tem acesso a este banco de dados. As categorias *atuante* , *candidato* e *clínico* dos associados foram consultadas. Esta lista foi compilada, e uma pesquisa no Google (Mountain View, CA, Estados Unidos) foi realizada usando o nome do membro + “trauma ortopédico” ou “cirurgião ortopédico” para determinar se o membro era um cirurgião atuante com treinamento especializado em traumatologia ortopédica. Apenas a primeira página de resultados de cada pesquisa foi visualizada. As informações acerca do treinamento especializado do cirurgião, com base em bolsa de estudos ou local de formação, foram usadas para determinar a inclusão no estudo. Os membros que não atendiam a esses critérios ou não tinham informações suficientes para a determinação desses dados foram excluídos.

Os dados demográficos coletados de cada membro incluíram nome, tipo de prática e local de atuação. Os locais de atuação foram agrupados em cinco regiões, sendo quatro nos Estados Unidos (Nordeste, Meio-oeste, Oeste e Sul), mais o Canadá. A Tabela 1 mostra a lista completa dos estados de cada região.

**Tabela 1:** Estados das regiões dos Estados Unidos

Regiões			
Oeste	Meio-oeste	Sul	Nordeste
Washington	Dakota do Norte	Texas	Maryland
Oregon	Dakota do Sul	Mississippi	Distrito de Colúmbia
Califórnia	Nebrasca	Arkansas	Pensilvânia
Idaho	Kansas	Louisiana	Delaware
Nevada	Oklahoma	Kentucky	Nova Jersey
Utah	Minnesota	Tennessee	Nova York
Arizona	Iowa	Missouri	Connecticut
Montana	Wisconsin	Alabama	Massachusetts
Wyoming	Illinois	Flórida	Rhode Island
Colorado	Michigan	Geórgia	Vermont
Novo México	Indiana	Carolina do Sul	New Hampshire
Alasca	Ohio	Carolina do Norte	Maine
Havaí	Virgínia Ocidental	Virgínia	
		Porto Rico	

## Análise das Redes Sociais

A análise de mídia social foi realizada de acordo com Narain et al. 3 e Lander et al. 5 Sete plataformas distintas foram avaliadas quanto à presença de membros: Facebook, X, Instagram, LinkedIn, ResearchGate, YouTube e *websites* profissionais. Esta avaliação utilizou um método padronizado, com os mesmos critérios da pesquisa anterior no Google em cada plataforma individual. Foram excluídos perfis pessoais ou privados que não estivessem primariamente relacionados à atuação profissional do cirurgião. O *website* deveria ter sido criado para esse cirurgião individual para que fosse incluído no estudo; a presença em *websites* departamentais ou de vários profissionais de um grupo foi excluída.

Após a identificação dos perfis nas plataformas, foi determinado o uso ativo de Facebook, X, Instagram e YouTube, definido como atividade no perfil nos últimos seis meses. As outras três plataformas (LinkedIn, ResearchGate e os *websites* profissionais) não foram avaliadas quanto à atividade porque não se baseiam em publicações ou atividades atuais. A pontuação geral de rede social foi calculada somando-se o número de perfis que cada membro apresentava em nossa pesquisa. Em relação às plataformas avaliadas quanto à atividade, apenas os perfis ativos foram contabilizados na pontuação de rede social.

## Análise Estatística

Os dados demográficos dos cirurgiões foram comparados. Variáveis categóricas foram expressas como frequências e porcentagens e comparadas pelo teste do qui-quadrado. As variáveis contínuas são expressas como médias com desvios padrão para variáveis com distribuição normal. Estes são comparados com o teste *t* de Student ou testes de análise de variância ( *analysis of variance* , ANOVA, em inglês). O alfa foi definido em 0,05. Todos as análises estatísticas e de dados foram realizadas usando o programa JMP Pro, versão 16.0 (SAS Institute Inc., Cary, NC, Estados Unidos).

## Resultados

## Dados Demográficos

Ao todo 1.262 membros foram identificados como cirurgiões atuantes com treinamento em traumatologia ortopédica: 90% (1.130) deles eram do sexo masculino, 567 (44,9%) atuavam em âmbito acadêmico, enquanto os outros 695 (55,1%) trabalhavam no setor privado. A região Sul continha o maior número de membros: 400 (31,7%). Os dados demográficos completos são mostrados na Tabela 2 .

**Tabela 2:** Dados demográficos dos cirurgiões

Variável	Cirurgiões ( *n* = 1.262)
**Sexo**	
Feminino	132 (10,5%)
Masculino	1.130 (89,5%)
**Âmbito de atuação**	
Acadêmico	567 (44,9%)
Setor privado	695 (55,1%)
**Região**	
Canadá	62 (4,9%)
Meio-oeste	272 (21,5%)
Nordeste	252 (20,0%)
Sul	400 (31,7%)
Oeste	276 (21,9%)

## Plataformas de Redes Sociais

A utilização das plataformas de rede social foi variável. O Instagram foi o menos prevalente, com apenas 34 (2,7%) membros com conta e 23 (1,8%) contas ativas. Em relação às contas atualmente ativas, o X foi o mais prevalente, com 91 (7,2%) contas, das quais 58 (4,6%) foram consideradas ativas. O LinkedIn foi a plataforma mais utilizada por mais da metade (54%) dos membros identificados com contas. A pontuação média de rede social foi de 1,24 ± 1,05 (intervalo: 0 a 6). Nenhum membro recebeu a pontuação perfeita de rede social de 7. A Tabela 3 mostra os detalhes sobre a utilização de redes sociais.

**Tabela 3:** Contas de mídia social por região

Plataforma	Total ( *n* = 1.262)	Canadá ( *n* = 62)	Meio-oeste ( *n* = 272)	Nordeste ( *n* = 252)	Sul ( *n* = 400)	Oeste ( *n* = 276)	Valor de *p*
Facebook	59 (4,7%)	0 (0%)	18 (6,6%)	8 (3,2%)	20 (5,0%)	13 (4,7%)	0,15
Ativas	30 (2,4%)	0 (0%)	8 (2,9%)	4 (1,6%)	12 (3,0%)	6 (2,2%)	0,52
X	91 (7,2%)	6 (9,7%)	21 (7,7%)	21 (8,3%)	27 (6,8%)	16 (5,8%)	0,73
Ativas	58 (4,6%)	4 (6,5%)	12 (4,4%)	17 (6,7%)	16 (4,0%)	9 (3,3%)	0,33
Instagram	34 (2,7%)	1 (1,6%)	8 (2,9%)	8 (3,2%)	11 (2,8%)	6 (2,2%)	0,93
Ativas	23 (1,8%)	1 (1,6%)	4 (1,5%)	5 (2,0%)	8 (2,0%)	5 (1,8%)	0,99
LinkedIn	683 (54,1%)	37 (59,7%)	145 (53,3%)	142 (56,3%)	218 (54,5%)	141 (51,1%)	0,65
ResearchGate	290 (23,0%)	20 (32,3%)	51 (18,8%)	71 (28,2%)	80 (20,0%)	68 (24,6%)	0,016
YouTube	433 (34,3%)	15 (24,2%)	96 (35,3%)	102 (40,5%)	137 (34,3%)	83 (30,1%)	0,056
*Website*	44 (3,5%)	1 (1,6%)	9 (3,3%)	6 (2,4%)	13 (3,3%)	15 (5,4%)	0,31
Pontuação de rede social	1,24 ± 1,05	1,26 ± 0,85	1,19 ± 1,03	1,38 ± 1,11	1,21 ± 1,04	1,18 ± 1,05	0,21

## Uso Regional de Redes Sociais

Dividido entre as quatro regiões dos Estados Unidos mais o Canadá, apenas a utilização da plataforma ResearchGate apresentou diferenças estatisticamente significativas ( *p* = 0,016). O Canadá liderou todas as regiões, com utilização desta plataforma por 32% dos cirurgiões, seguido do Nordeste dos Estados Unidos, com 28%. Detalhes da utilização da plataforma de rede social são mostrados na Tabela 3 .

## Tipo de Atuação e Uso de Redes Sociais

Houve variação com relação ao uso das redes sociais por cirurgiões nas práticas acadêmica e privada em algumas plataformas. Os cirurgiões acadêmicos estavam mais propensos a ter uma conta ativa no X ( *p* = 0,0001) e uma página no LinkedIn ( *p* = 0,0055), no ResearchGate ( *p* < 0,0001) e no YouTube ( *p* = 0,0002). Os cirurgiões particulares estavam mais propensos a ter um *website* profissional ( *p* = 0,0008). Os cirurgiões acadêmicos apresentaram maior pontuação média de rede social (1,43) do que os cirurgiões particulares (1,08) ( *p* < 0,0001) ( Tabela 4 ).

**Tabela 4:** Contas de mídia social por tipo de atuação

Plataforma	Total ( *n* = 1262)	Acadêmico ( *n* = 567)	Setor privado ( *n* = 695)	RR (IC95%)	Valor de *p*
Facebook	59 (4,7%)	20 (3,5%)	39 (5,6%)	0,627 (0,37–1,06)	0,084
Ativas	30 (2,4%)	9 (1,6%)	21 (3,0%)	0,52 (0,24–1,14)	0,1
X	91 (7,2%)	55 (9,7%)	36 (5,2%)	1,87 (1,25–2,8)	0,0025
Ativas	58 (4,6%)	42 (7,4%)	16 (2,3%)	3,21 (1,83–5,65)	0,0001
Instagram	34 (2,7%)	17 (3,0%)	17 (2,4%)	1,01 (0,99–1,02)	0,55
Ativas	23 (1,8%)	13 (2,3%)	10 (1,4%)	1,59 (0,7–3,6)	0,27
LinkedIn	683 (54,1%)	331 (58,4%)	352 (50,6%)	1,15 (1,04–1,28)	0,0055
ResearchGate	290 (23,0%)	184 (32,5%)	106 (15,3%)	2,12 (1,72–2,63)	< 0,0001
YouTube	433 (34,3%)	225 (39,7%)	208 (29,9%)	1,33 (1,14–1,55)	0,0002
*Website*	44 (3,5%)	8 (1,4%)	36 (5,2%)	0,27 (0,13–0,58)	0,0008
Pontuação de rede social	1,24 ± 1,05	1,43 ± 1,10	1,08 ± 0,97		< 0,0001*

Abreviaturas: IC95%, intervalo de confiança de 95%; RR, risco relativo.

Nota: *teste *t* .

## Discussão

Este é o primeiro estudo a examinar o uso das redes sociais entre traumato-ortopedistas. Observamos que os traumato-ortopedistas subutilizam as redes sociais. O uso não variou de forma significativa com relação aos locais de atuação, mas foi diferente entre cirurgiões acadêmicos e privados, estando os acadêmicos mais propensos a usar diversas plataformas e ter pontuações gerais maiores.

Tradicionalmente, os pacientes dependiam das recomendações de outros médicos e do boca a boca para indicações de especialistas e cirurgiões. Na última década, o uso de redes sociais aumentou 10 vezes, e cerca de 80% dos usuários relatam fazer pesquisas na internet sobre médicos, doenças ou opções de tratamento. 2 À medida que a população depende cada vez mais das redes sociais e das pontuações nas escalas de satisfação dos pacientes para a escolha dos profissionais de saúde, as estratégias de autopromoção e envolvimento dos pacientes terão de ser adaptadas para abarcar estas novas plataformas.

Em uma análise do uso das redes sociais em cirurgia plástica, descobriu-se que uma só postagem pode gerar de 10 a 12 repostagens, o que amplia o alcance relativo à autopromoção. 6 7 Pesquisas sobre outras subespecialidades ortopédicas demonstraram que a presença *on-line* mais robusta estava associada a pontuações maiores nas escalas de satisfação do paciente. 8 9 Neste estudo, a pontuação média nas redes sociais encontrada entre os traumatologistas foi de 1,24 ± 1,05 (intervalo: 0 a 6). Donnally et al. 8 relataram que cirurgiões de mão com pontuação de mídia social inferior a 3 receberam avaliações mais baixas no Healthgrades do que aqueles que tiveram índice de mídia social acima de 3. Isso destaca o potencial para traumato-ortopedistas melhorarem a satisfação e o envolvimento do paciente por meio das redes sociais. Mais pesquisas são necessárias para compreender o impacto das redes sociais no crescimento da prática ortopédica e na satisfação do paciente com a traumatologia ortopédica.

Enfatizando ainda mais a crescente relevância das redes sociais na prática ortopédica, a American Academy of Orthopaedic Surgeons (AAOS), bem como o *Journal of Orthopaedic Trauma* , publicaram diretrizes e recomendações para o desenvolvimento da presença nas redes sociais. 2 9 10 Esses recursos incentivam os médicos a participarem em redes sociais para maximizar oportunidades ou para o seu desenvolvimento pessoal e profissional. As diretrizes descrevem os princípios básicos de uso para iniciantes em redes sociais e dão orientações sobre como manter o padrão profissional no espaço digital.

Nosso estudo demonstrou que os traumato-ortopedistas utilizam pouco as plataformas de mídia social mais populares. Entre os cirurgiões, 34,3% tinham presença ativa no YouTube, 2,4% tinham uma página ativa no Facebook e 1,8% tinham uma conta ativa no Instagram. Esses números representam uma taxa de uso substancialmente menor em comparação às médias de uso de redes sociais por adultos nos Estados Unidos, de 73% no YouTube, 69% no Facebook e 37% no Instagram. 1 3 A subutilização das redes sociais também foi relatada em outras áreas da cirurgia ortopédica. Dos 987 cirurgiões ortopédicos pediátricos, 33% tinham presença no YouTube e 14% tinham uma página profissional no Facebook. 5 Na cirurgia do ombro e cotovelo, 12,9% dos cirurgiões pesquisados tinham presença ativa no YouTube, e 10,4% tinham uma página profissional no Facebook. 3 A baixa utilização das redes sociais entre traumato-ortopedistas representa a perda de uma oportunidade de prática e desenvolvimento profissional.

O local de atuação tende a influenciar os padrões clínicos gerais, especialmente em relação à autopromoção e ao encaminhamento de pacientes. Embora os traumato-ortopedistas do âmbito acadêmico utilizem as redes sociais de forma mais extensiva do que seus pares em clínicas particulares neste estudo, isso contradiz muitas outras pesquisas sobre o uso dessas redes na área da saúde, que é crescente no ambiente privado. 4 6 11 Isso pode ser devido à menor necessidade de uma base de indicações e autopromoção em traumatologia em comparação a outras práticas cirúrgicas. Na traumatologia ortopédica, o aumento do uso das redes sociais em um ambiente acadêmico pode estar relacionado à sua utilização principalmente como uma ferramenta educacional, não como um meio de recrutamento de pacientes. Estudos demonstraram associações entre a produtividade acadêmica e o aumento do uso das redes sociais. 12 13

O baixo envolvimento nas redes sociais entre os traumato-ortopedistas pode ter vários motivos. Os cirurgiões podem considerar a rede social um meio ineficiente, especialmente se não tiverem experiência no uso das diversas plataformas. 14 Além disso, pode haver menos motivação para a utilização das redes sociais na traumatologia em comparação a outras subespecialidades ortopédicas eletivas que dependem mais da escolha de um profissional de saúde pelo paciente e uma sólida base de indicações. 12 A utilização das redes sociais nos cuidados de saúde não é isenta de controvérsia ou preocupação. Os cirurgiões podem estar preocupados com a possibilidade de violações da Lei de Portabilidade e Responsabilidade de Seguros de Saúde (HIPAA, do inglês Health Insurance Portability and Accountability Act) de 1996 e com a manutenção do profissionalismo ou dos padrões éticos. Além disso, também pode haver preocupações quanto à capacidade de manter limites no contato médico-paciente. 2 Também pode haver baixo uso devido a preocupações relacionadas à reputação pessoal e ao profissionalismo. Mais de 60% dos recrutadores verificam as contas de redes sociais de potenciais funcionários. 10 De modo geral, as redes sociais podem trazer benefícios para a prática de traumatologia e ortopedia, incluindo *networking* profissional, educação e envolvimento do paciente. No entanto, é essencial que os traumato-ortopedistas sigam um padrão rigoroso de profissionalismo ao integrar as redes sociais à sua prática clínica.

Este estudo tem diversas limitações. Primeiro, analisamos apenas membros da OTA com informações públicas relativas ao seu treinamento e que mantêm contas de domínio público nas redes sociais. Portanto, nossos resultados podem não ser completamente representativos do uso das redes sociais entre os traumatologistas como um todo. Além disso, este estudo foi de natureza observacional, e representa um determinado momento. O uso das redes sociais é dinâmico e o momento avaliado pode subestimá-lo ou superestimá-lo. Mais pesquisas são necessárias para compreender o tipo de conteúdo e materiais que facilitam a educação, o *networking* e o envolvimento do público. Por último, neste estudo, não pudemos comentar sobre as barreiras ou preocupações acerca do uso das redes sociais entre os traumato-ortopedistas.

## Conclusão

Atualmente, as plataformas de rede social são subutilizadas por traumato-ortopedistas. Mais pesquisas são necessárias para compreender as barreiras à utilização das redes sociais na comunidade da OTA.
